# Radiogenomics of lower-grade gliomas: a radiomic signature as a biological surrogate for survival prediction

**DOI:** 10.18632/aging.101594

**Published:** 2018-10-22

**Authors:** Zenghui Qian, Yiming Li, Zhiyan Sun, Xing Fan, Kaibin Xu, Kai Wang, Shaowu Li, Zhong Zhang, Tao Jiang, Xing Liu, Yinyan Wang

**Affiliations:** 1 Beijing Neurosurgical Institute, Capital Medical University, Beijing, China; 2 Department of Neurosurgery, Beijing Tiantan Hospital, Capital Medical University, Beijing, China; 3 Department of Neuroradiology, Beijing Tiantan Hospital, Capital Medical University, Beijing, China; 4 Department of Nuclear Medicine, Beijing Tiantan Hospital, Capital Medical University, Beijing, China; 5 Center of Brain Tumor, Beijing Institute for Brain Disorders, Beijing, China; 6 Chinese Academy of Sciences, Institute of Automation, Beijing, China; * Equal contribution

**Keywords:** gliomas, prognosis, genetics, magnetic resonance imaging, biomarkers

## Abstract

Objective: We aimed to identify a radiomic signature to be used as a noninvasive biomarker of prognosis in patients with lower-grade gliomas (LGGs) and to reveal underlying biological processes through comprehensive radiogenomic investigation. Methods: We extracted 55 radiomic features from T2-weighted images of 233 patients with LGGs (training cohort: *n* = 85; validation cohort: *n* = 148). Univariate Cox regression and linear risk score formula were applied to generate a radiomic-based signature. Gene ontology analysis of highly expressed genes in the high-risk score group was conducted to establish a radiogenomic map. A nomogram was constructed for individualized survival prediction.

Results: The six-feature radiomic signature stratified patients in the training cohort into low- or high-risk groups for overall survival (*P* = 0.0018). This result was successfully verified in the validation cohort (*P* = 0.0396). Radiogenomic analysis revealed that the prognostic radiomic signature was associated with hypoxia, angiogenesis, apoptosis, and cell proliferation. The nomogram resulted in high prognostic accuracy (C-index: 0.92, C-index: 0.70) and favorable calibration for individualized survival prediction in the training and validation cohorts.

Conclusions: Our results suggest a great potential for the use of radiomic signature as a biological surrogate in providing prognostic information for patients with LGGs.

## Introduction

Diffuse lower-grade gliomas (LGGs) are a class of terminal central nervous system tumors, comprising WHO grades II and III astrocytomas, oligodendrogliomas, and mixed oligoastrocytomas [1,2]. Although maximal safe resection combined with adjuvant radiotherapy and chemotherapy shows prognostic benefits [3], the overall survival of patients with LGGs remains low, ranging from a few months to more than 10 years [4,5]. This demonstrates the need for in depth investigations of prognosis of patients with LGGs.

Developments in genomic and bioinformatic techniques allow the creation of molecular classifications and signatures based on expression profiles, and provide promising approaches to identify prognostic or therapeutic biomarkers for patient-tailored management [2,6,7]. In a recent study, utilization of IDH mutation and 1p/19q codeletion, LGGs could be classified into 3 distinct subgroups (IDH wild-type, IDH mutation and 1p/19q codeletion, and IDH mutation and 1p/19q non-codeletion) that capture the biologic characteristics with greater fidelity than does histological class [2]. Although such genetic characteristics can be informative and are relatively homogeneous within each tumor, there remains an unmet clinical need for less costly and less time consuming noninvasive surrogates able to determine clinical prognostic and guide individual treatment.

MRI can provide a comprehensive view of the entire tumor, and is routinely used as a noninvasive tool to support clinical decision-making, histological grading, and therapeutic monitoring [8,9]. Radiomics, an emerging field that extracts a large number of quantitative descriptors reflecting textural and morphological variations, has been introduced to ensure more objective and precise study of oncologic tissue beyond established MRI metrics [10-12]. Consequently, clinicians increasingly rely on radiomics to assist in personalizing treatment in clinical practice, particularly in relation to tumor detection, subtype classification, and prognostic estimation [13-15]. Furthermore, linking radiomics with genomic characteristics, i.e., radiogenomics, has become an increasingly popular approach in various tumors and has expanded to create non-invasive imaging biomarkers for genomic aberrations [16-19]. As for LGGs, Mazurowski et al. [20] have done a preliminary radiogenomics study focusing on the relationship between tumor shape and molecular subtype in a single cohort. However, this study was limited by small number of available radiomic features and the lack of an external validation cohort.

In the present study, we used T2-weighted MR images from The Cancer Imaging Atlas (TCIA) to identify a radiomic-based prognostic signature, which we independently validated in the Chinese Glioma Genome Atlas (CGGA) imaging dataset. A radiogenomic map, which integrates radiomic features and genomic data, was further established to identify biological processes underlying this radiomic signature. Our results suggest that radiomics can aid in predicting survival of patients with LGGs, and reveal the prognostic role of radiomic phenotypes using comprehensive radiogenomic methods.

## RESULTS

### Prognostic value of the radiomic risk score

**Figure 1:**
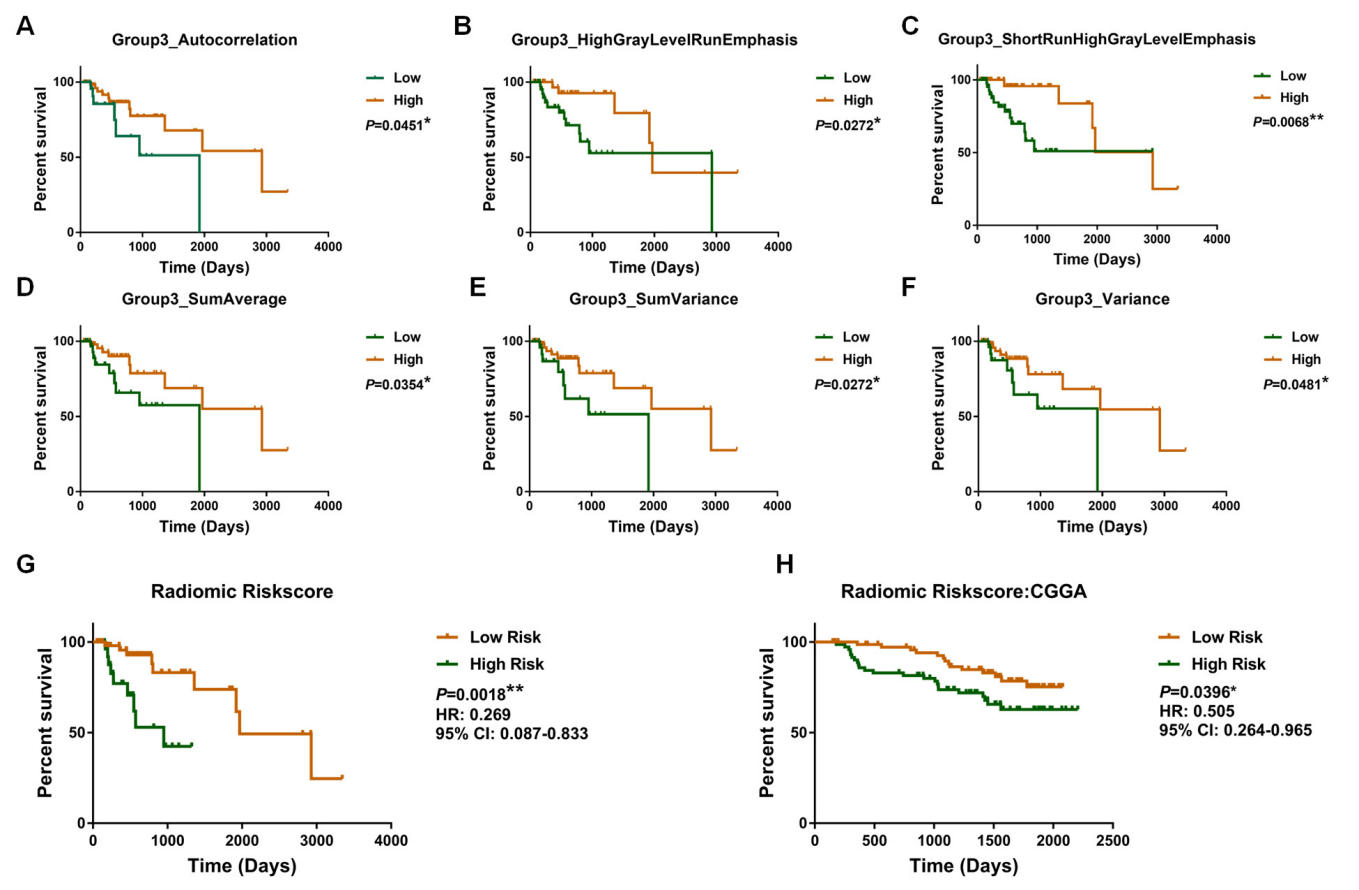
Kaplan–Meier plot for overall survival of patients stratified by the value of each radiomic feature (**A**, **B**, **C**, **D**, **E**, **F**) and radiomic risk score (**G**) in the training dataset. The radiomic risk score retained prognostic significance for patients in the validation set (**H**).

We found that radiomic features (Autocorrelation, High Gray Level Run Emphasis (HGLRE), Short Run High Gray Level Emphasis (SRHGLE), SumAverage, SumVariance, and Variance) were significantly associated with overall survival (Supplementary Table 1). Moreover, we observed that each of the selected radiomic features could be used to stratify patients into high-risk and low-risk groups (Autocorrelation, *P* = 0.0451; HGLRE, *P* = 0.0272; SRHGLE, *P* = 0.0068; SumAverage, *P* = 0.0354; SumVariance, *P* = 0.0272; and Variance, *P* = 0.0281; Fig. 1A-F).

Subsequently, a radiomic risk score were calculated: risk score = Autocorrelation × (-0.007) + HGLRE × (-0.003) + SRHGLE × (-0.005) + SumAverage × (-0.115) + SumVariance × (-0.002) + Variance × (-0.007). The radiomic risk score was associated with overall survival in the training dataset (*P* = 0.00018; HR = 0.269, 95% confidence interval [CI]: 0.087–0.833; Fig. 1G).

Consistently, we confirmed the prognostic value of selected radiomic features in the validation dataset (Autocorrelation, *P* = 0.0081; HGLRE, *P* = 0.0120; SRHGLE, *P* = 0.0085; SumAverage, *P* = 0.0168; SumVariance, *P* = 0.0058; and Variance, *P* = 0.0063; Supplementary Fig. 1), as well as confirming the prognostic value of the radiomic risk score (*P* = 0.0396; HR = 0.505; 95%CI: 0.264–0.965; Fig. 1H).

We next conducted multivariate Cox regression analyses in TCGA database, which indicated that the radiomic risk score was an independent prognostic factor (P = 0.042). Other independent prognostic factors were age, WHO grade, and IDH status. The prognostic value of all clinical characteristics in the multivariate Cox regression analyses are shown in Table 1.

**Table 1:** Clinical characteristics of lower grade gliomas in TCGA and CGGA datasets.

	**TCGA cohort (n=85)**	**CGGA cohort (n=148)**
**Age (range, median)**	20-74(43)	18-63(38)
**Sex**		
Female	49	54
Male	36	94
**WHO Grade**		
WHO II	45	105
WHO III	40	43
**Seizure**		
Yes	56	89
No	29	59
**IDH status**		
Mutant	65	109
Wildtype	20	39
**ATRX**		
Mutant	34	NA
Wildtype	51	NA
**1p/19q**		
Codeletion	21	22
Non-codeletion	64	47
NA	0	79

NA = Not Available; TCGA = the Cancer Genome Atlas; CGGA = Chinese Glioma Genome Atlas.

### Functional annotation of different prognoses

**Figure 2:**
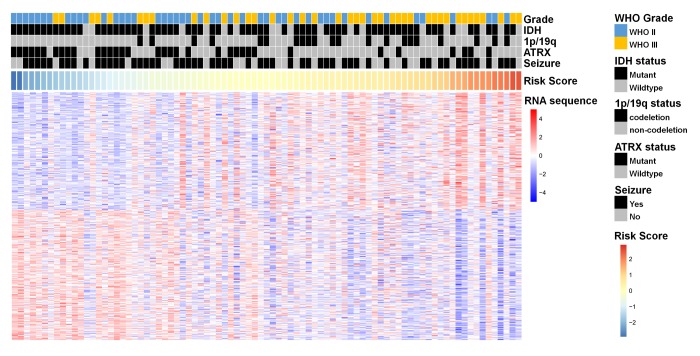
A heat map of the top 200 genes that were positively associated with the radiomic risk score (upper half part) and the top 200 genes that were negatively associated with the radiomic risk score (lower half part) from 85 LGGs samples in the training dataset. “RNA sequence” refers to the overall expression levels of the genes. Associations of clinicopathological characteristics with radiomic features are illustrated.

**Figure 3:**
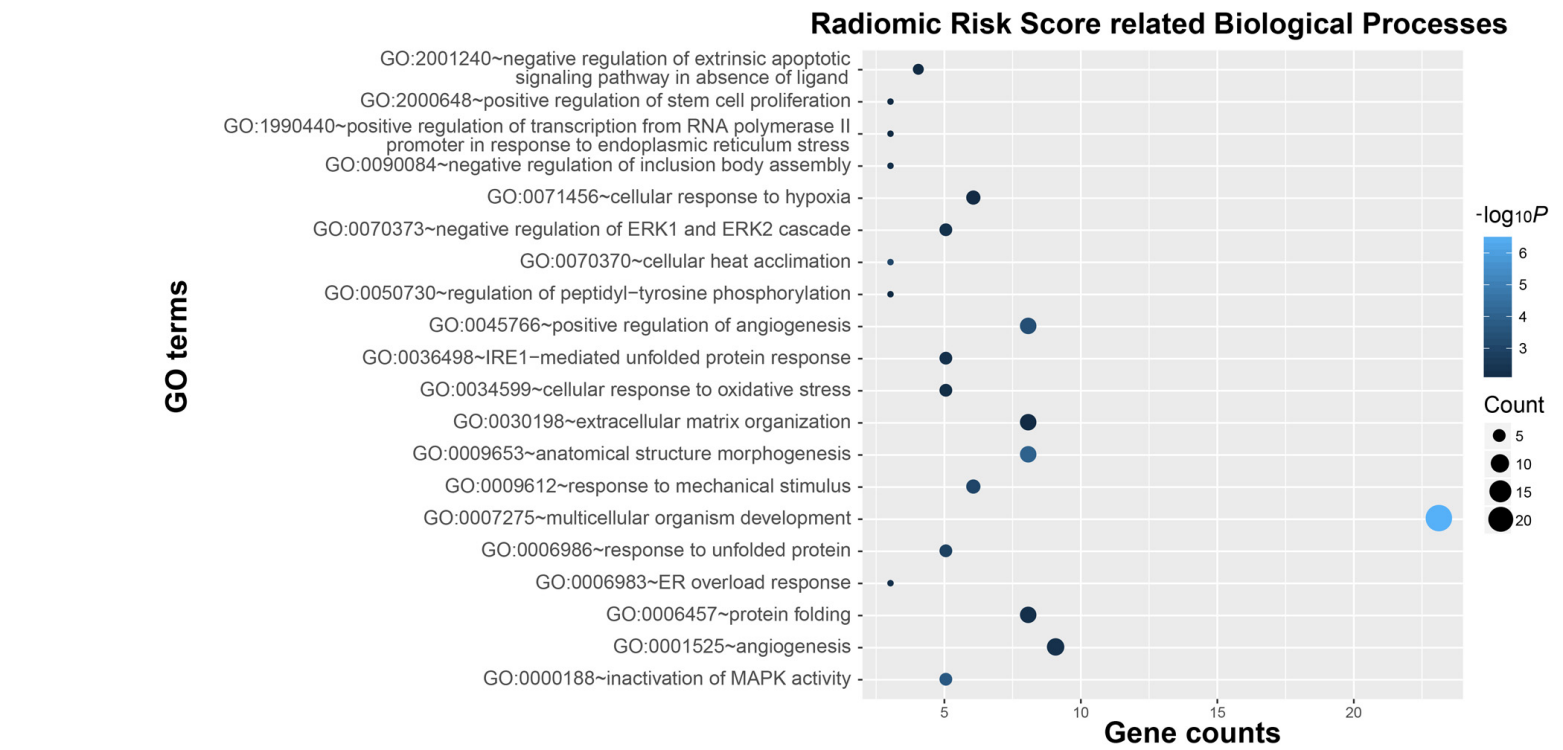
Functional annotation of radiomic risk score groups. Gene ontology analysis revealed a significant association among genes with increased expression in the high-risk radiomic risk score group and twenty main pathways. Column size: gene counts; point color: enrichment *P* value.

To explore the genetic background of prognostic differences, relevant transcriptomic profiles were analyzed. The radiogenomic analysis of high-risk positively associated genes (*n* = 239, Fig. 2) further revealed that biological processes associated with prognosis included hypoxia, angiogenesis, and stem cell proliferation-related oncogenic functions (Fig. 3). Specifically, genes in the “multicellular organism development” group are the ones that are most significantly associated to the radiomic risks score. Further investigation revealed that SPRED1 and SPRED2 were the most correlated genes involved in “multicellular organism development” (Supplementary Table 2).

Similar findings were obtained during the assessment of genetic alterations underlying the six texture features (Supplementary Fig. 2). As shown in Supplementary Fig. 3, the radiomics-based evaluation may stand for patients with different expression profiles and biological functions among the three molecular classification, therefore serving as a supplementary approach for tailored medicine of LGGs.

### Construction of individualized prediction models

**Figure 4:**
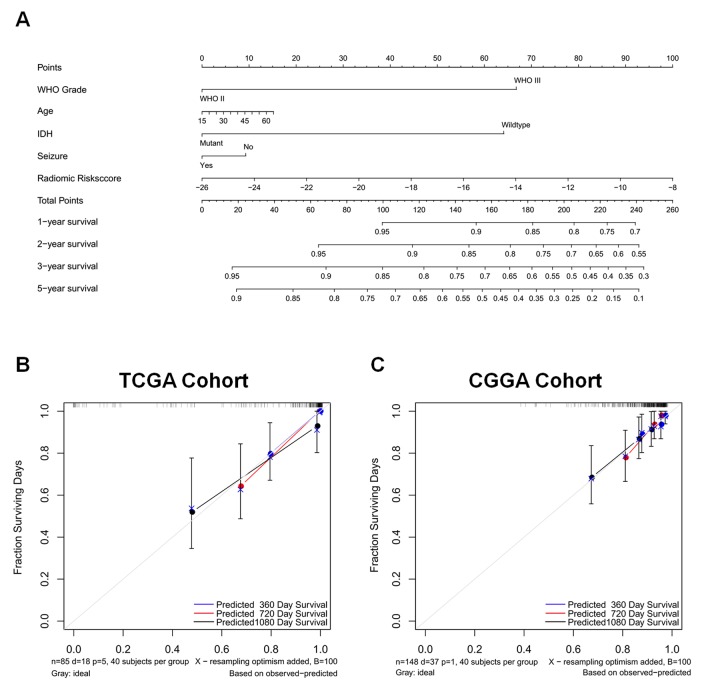
A nomogram for predicting overall survival of patients with LGGs (**A**), along with the assessment of model calibration in the training cohort (**B**) and validation cohort (**C**). After final model selection, radiomic signature, WHO grade, age, IDH status, and seizure were included in the nomogram. The line determines the number of points received for the value of each variable. The sum of these numbers is presented on the total axis, while the line drawn down to the survival axis determines the likelihood of a 1-, 2-, 3-, or 5-year survival rate. The calibration curve of the nomogram is also shown. Three colored lines (blue, red, and black) represent the performance of the nomogram, with a closer fit to the diagonal line representing a better estimation.

The independent prognostic parameters for overall survival in the training cohort, including WHO grade, age at diagnosis, IDH, seizure, ATRX, and radiomic risk score, were integrated into the nomogram (Supplementary Fig. 4). The C-index of the nomograms for overall survival was 0.934. Meanwhile, the calibration plot for the probability of survival showed optimal agreement. Since the ATRX status for patients with LGG was not available in the validation cohort, a prognostic nomogram that integrated all factors except for ATRX was constructed in the training cohort and independently validated in the validation cohort. The C-indices were 0.92 and 0.70 in the training cohort and validation cohort, respectively, indicating satisfactory concordance. Moreover, the calibration plots of the probability of actual survival were concordant with survival outcomes predicted by the nomograms at 1, 2, and 5 years for overall survival (Fig. 4).

## DISCUSSION

In the present study, we revealed a radiomic-based signature that noninvasively predicted survival in both the training (TCIA) and validation (CGGA) cohorts. Integrative analysis of radiomic and transcriptomic profiles suggested that a high-risk phenotype indicated by the radiomic analysis could be attributed to several malignant biological processes. Moreover, the combination of radiomic, clinical, and molecular risk factors into a nomogram provided an effective approach for individual survival estimation.

Radiomics is a promising paradigm for extending clinical imaging into comprehensive and quantitative features, and has attracted much interest in personalized medicine [21,22]. Few studies have identified radiomics could be reliable prognostic biomarkers for stratification of patients across many fields of oncology, however, the currently available studies are typically characterized by smaller sample sizes or lack of using independent public database as validation cohort [12,23,24]. Our analyses expand on the work of several recent studies that have uncovered novel associations among radiomic features and several clinical endpoints. The identified radiomic signature in our study consisted of the following features: Autocorrelation, HGLRE, SRHGLE, SumAverage, SumVariance, and Variance, which were derived from the group (iii) parameters (gray level co-occurrence and gray level run-length texture matrices-based parameters). We believe that these features describe textural differences based on gray-tone spatial dependencies, as opposed to relationships or patterns between pixels derived from first-order statistics. Such features provide further insight into tissue microstructure and the local environment of the tumor. Being consistent with our findings, a previous study reported that the most dominant prognostic features are derived from group (iii) [25].

Although each of the identified radiomic features in our study is capable of risk stratification, an integrated signature based on these textures achieved a better performance. Pioneering studies supported our findings that a multi-component radiomic signature provides a more statistically robust approach, and have consistently demonstrated the incremental value of the radiomic signature for risk stratification of patients with different cancers [25,26]. Notably, recent evidence has supported the hypothesis that radiological manifestations are tightly connected with genetic alterations of the tumor [27,28]. In the present study, the high-risk phenotype was significantly associated with oncogenic biological processes, including stem cell proliferation and angiogenesis, which could partially represent the malignancy of high-risk LGGs. As a classical hallmark of tumors, angiogenesis has emerged as a promising target for individually tailored medicine, although the therapeutic efficacy of anti-angiogenetic agents in gliomas has proved unsatisfactory [29]. This failure was attributed to ambiguities regarding the appropriate population for such treatment. Our results suggest a radiological indicator for vigorous angiogenesis in gliomas, thereby promoting the clinical effectiveness of anti-angiogenic therapy. Therefore, clinical trials exploring the relationship between radiological manifestation and target therapy are urgently needed to elucidate the possible implications of translating radiomic signatures into clinical practice. Additionally, “multicellular organism development” genes were a group of genes that were found to be highly associated with the radiomic risk score. SPRED1 and SPRED2 are the two most relevant genes and are members of the SPRED family of proteins that regulate growth factor-induced activation of the MAP kinase cascade [30]. The mechanisms of the high association of multicellular organism development with the radiomic risk score are still to be investigated.

The objective of precision medicine is to build a model that offers a more tailored approach for individual patients considering their individual prognostic factors. A nomogram is a graphical representation of a statistical model that allows individualized predictions and compares favorably to traditional risk grouping systems. Incorporating the radiomic signature into a nomogram along with clinicopathologic risk factors to estimate disease-free survival for early-stage non–small cell lung cancer has provided better estimation than either the radiomic signature or clinicopathologic nomogram alone [31]. The new WHO classification of the central nervous system has improved the clinical assessment of LGGs [32]. Indeed, our classifier will be more relevant if it incorporates those prognostics genomic subtypes. However, in practice, not all genomic information (e.g., 1p/19q) is available for all patients; hence, classifier should also be designed to accommodate sparse data. In general, we developed a nomogram in the training cohort by incorporating WHO grade, age at diagnosis, IDH, seizure, and radiomic risk score. Those prognostic factors were independent prognostic factors with using multivariate Cox regression analysis, suggesting their complementary value in predicting LGGs prognosis. The higher degree of predictive precision in our nomogram could be attributed to the integration of different dimensions of information (genetics, clinical, and imaging data), which provides a complementary perspective about a single tumor. In the validation cohort, age and seizure lost their prognostic power in a multivariate model, which may explain for the decreased performance of the prognostic model. Despite this, our prognostic model still displayed good accuracy (CI=0.7) in the external validation cohort, which has strengthened the reliability of the results. Our findings are consistent with the perspective that future research will be most productive if we concentrate on populating a three-domain Venn diagram intersection consisting of imaging, genetics, and clinical data [33].

There are several limitations that should be considered when interpreting the results of this study. First, as a multi-center study, the imaging data used were acquired from multiple MRI systems with varying protocols. Better image quality and consistent protocols will further improve the power of radiomics. Second, the radiomics analysis was performed using T2-weighted MR images in the current study. Although the FLAIR sequence is favorable for lesion delineation it was not used in the radiomics analysis due to limited availability of FLAIR data in the CGGA image database. Third, a slight discrepancy in the manual segmentation of images into the region of interest (ROI) may still exist, even though the image segmentation was carefully controlled by 2 radiologists. The predictive value of radiomic signatures need to be further characterized and validated in prospective study using multi-modal imaging approaches.

## CONCLUSIONS

In conclusion, this radiogenomic study established a radiological indicator for prognostic assessment in patients with LGGs. The biological processes associated with the radiomic features was further revealed. The findings of this study may be an important aid to the decision making in personalized clinical management for glioma patients.

## MATERIALS AND METHODS

### Patient selection

There were a total of 233 patients enrolled in this study (Supplementary Table 3), including 85 from The Cancer Genome Atlas (TCGA, training cohort) and 148 patients from CGGA (validation cohort). Selection criteria for both cohorts included: (a) histopathologically confirmed grade II or III gliomas, according to WHO classification [32]; (b) minimum age of 18 years; and (c) available preoperative T2-weighted MR images. Specifically, MRI data were available for 199 lower-grade glioma patients in TCGA database. A set of 103 cases without high-quality preoperative T2 images and 11 cases without overall survival data were excluded. Thus, there were 85 lower-grade glioma cases that were included in the current study. Baseline epidemiologic and clinical characteristics of all patients, including age, sex, seizure, WHO grade, and molecular status, are shown in Table2. This retrospective study protocol was approved by the ethics committee of Beijing Tiantan Hospital. All clinical data and biological information were collected based on published databases.

**Table 2:** Variables associated with overall survival in the Cox regression analysis for lower-grade glioma patients from the TCGA dataset.

	**Multivariate Cox Regression**		
	**HR**	**95% CI**	***P* value**
**Age**			
>45 vs. ≤45	5.788	1.024-32.709	**0.047**
**Sex**			
Male vs. Female	0.500	0.148-1.691	0.265
**WHO Grade**			
III vs. II	22.499	1.913-264.626	**0.013**
**Seizure**			
Yes vs. No	0.304	0.089-1.036	0.057
**IDH status**			
WT vs. MUT	27.578	2.816-270.110	**0.004**
**ATRX status**			
WT vs. MUT	0.221	0.042-1.164	0.075
**1p/19q status**			
Non-codel vs. codel	2.117	0.156-28.724	0.573
**Radiomic Risk score**			
High vs. Low	4.347	1.055-17.922	**0.042**

MUT = mutant. WT, wild type; Non-codel = non-codeletion; Codel = codeletion; HR = hazard ratio; 95% CI = 95% confidence interval.

### Image acquisition and tumor segmentation

MR images in the training cohort were collected from TCIA dataset (http://www.cancerimagingarchive.net). MR images in the validation cohort were obtained from the CGGA imaging database (http://www.cgga.org.cn), and the image acquisition was performed as described in our previous publication [34]. Lesions were delineated on T2-weighted images, as T2-weighted MRI is a widely applied sequence for lesion characterization of brain diseases, specifically for brain tumors. In the majority of studies investigating LGGs, T2 MR imaging was well accepted in the identification of abnormal signal intensity representing the involved regions of LGGs.

Images from both cohorts underwent the same preprocessing procedures. To achieve reliable segmentation, manual segmentation was applied in the radiomic analysis in this study [15,24,26,35]. The ROI was manually drawn from the T2-weighted images abnormality on each slice by two experienced neuroradiologists (J.M. and X.C., both with more than 15 years of experience of diagnosis) by using MRIcro (http://www.mccauslandcenter.sc.edu/mricro/). In cases of a discrepancy of more than 5% (Dice index < 95%) in the tumor border outlines between the neuroradiologists, a third senior neuroradiologist (S.L. with more than 20 years of experience) made the final decision.

### Extraction of radiomic features

In order to avoid of the bias from data heterogeneity, all MRI data were normalized (Z score transformation with excluding segmented tumor areas) and re-sampled to the same resolution before feature extraction with using an in-house MATLAB process. Fifty-five quantitative radiomic features were extracted from the ROI using an automated method as previously described [25]. The features can be divided into three groups: (i) first-order statistics; (ii) shape and size based features; and (iii) textural features. In group (i), we estimated 14 texture parameters describing the distribution of voxel intensities within the ROI. In group (ii), we estimated eight 3D features describing 3D size and shape of the tumor region. In group (iii), we estimated 33 textural features describing patterns or spatial distribution of voxel intensities, which can provide information regarding the relative position of various gray levels over the image with gray level co-occurrence and gray level run-length texture matrices. All features were extracted using MATLAB 2014a (Mathworks, Natick, United States), and are listed in Supplementary Table 4.

### RNA sequencing and biomarker detection

Whole genome RNA sequencing data and relevant clinical and molecular neuropathological information were downloaded from the TCGA database (http://cancergenome.nih.gov/). The RNA sequencing data was normalized using the fragments per kilobase transcriptome per million reads method [36]. Transcriptome data were collected to identify potential biological processes underlying the radiomic signature. Presence of IDH mutation, 1p/19q codeletion, and ATRX mutation were also collected. For the validation cohort, IDH mutations were detected with pyrosequencing and Chromosome 1p/19q status were inferred by a Gaussian window smoothing algorithm using the expression values of the genes located on Chr-1p and Chr-19q, which have been described previously [37,38].

### Construction of the radiomic risk score

To obtain prognostic radiomic features in LGGs, we applied univariate Cox regression analyses of 55 features in the training dataset. Subsequently, the selected imaging features (*P* <0.05) were used to develop a radiomic signature. To investigate the effectiveness of the radiomic signature for clinical outcome prediction, a radiomic risk score was computed for each patient by linearly combining the selected features weighted by their corresponding coefficients as follows:

Risk score = expr_feature1_ × β_feature1_ + expr_feature2_ × β_feature2_ + … + expr_feature n_ × β_feature n_.

The same β values were applied to the validation cohort.

### Prediction of survival outcome using radiomic risk scores

Patients with LGG in the training and validation cohorts were divided into high-risk and low-risk groups referring to the median value of the radiomic risk score. The potential association of the radiomic risk score with overall survival was first assessed in the training dataset and then validated in the validation dataset with Kaplan-Meier survival analysis. Similarly, the prognostic value of each feature in the risk score was also evaluated based on Kaplan-Meier survival analysis. Patients were classified into “high” and “low” groups referring to the median value of each radiomic feature. Multivariate Cox regression analysis was performed to identify if the radiomic risk score is an independent prognostic factor.

### Radiogenomic analysis

Genes with significant associations from the radiomic-based signature or each radiomic feature were selected with Pearson correlation analysis, conducted with R programming language (http://cran.r-project.org). The candidate genes that positively related to radiomic risk score among the three molecular classification were selected with Pearson correlation analysis. Those with *P* <0.05 and a Pearson correlation coefficient >0.3 were considered as significant associated candidates. Positively-associated genes were then subjected to DAVID (http://david.ncifcrf.gov/) based gene ontology analysis to identify underlying biological processes. Biological processes with *P* <0.05 were depicted using the *ggplot2* package of R.

### Individualized prediction model construction

To establish a model that can predict an individual patient’s overall survival, a nomogram was formulated based on the results of the multivariate analysis with the *rms* package in R [39]. The final model was constructed with a backward stepdown selection process conforming to the Akaike information criterion [40]. Concordance index (C-index) and calibration curves were used to measure the predictive accuracy and discriminative ability of the nomograms. During the external validation of the nomogram, the total score (according to the nomogram) of each patient in the validation cohort were calculated and used as a factor in Cox regression analysis, and the C-index and calibration curve were then obtained [41].

## Supplementary Material

Supplementary Figures

Supplementary Tables
