# Supplementary material for: Radiogenomics of lower-grade gliomas: a radiomic signature as a biological surrogate for survival prediction
Source: Aging (Albany NY). 2018 Oct 22;10(10):2884–99. doi: 10.18632/aging.101594 (PMC6224242; doi:10.18632/aging.101594)
Supplement: Supplementary Figures [file aging-10-101594-s001.pdf]

SUPPLEMENTARY FIGURES

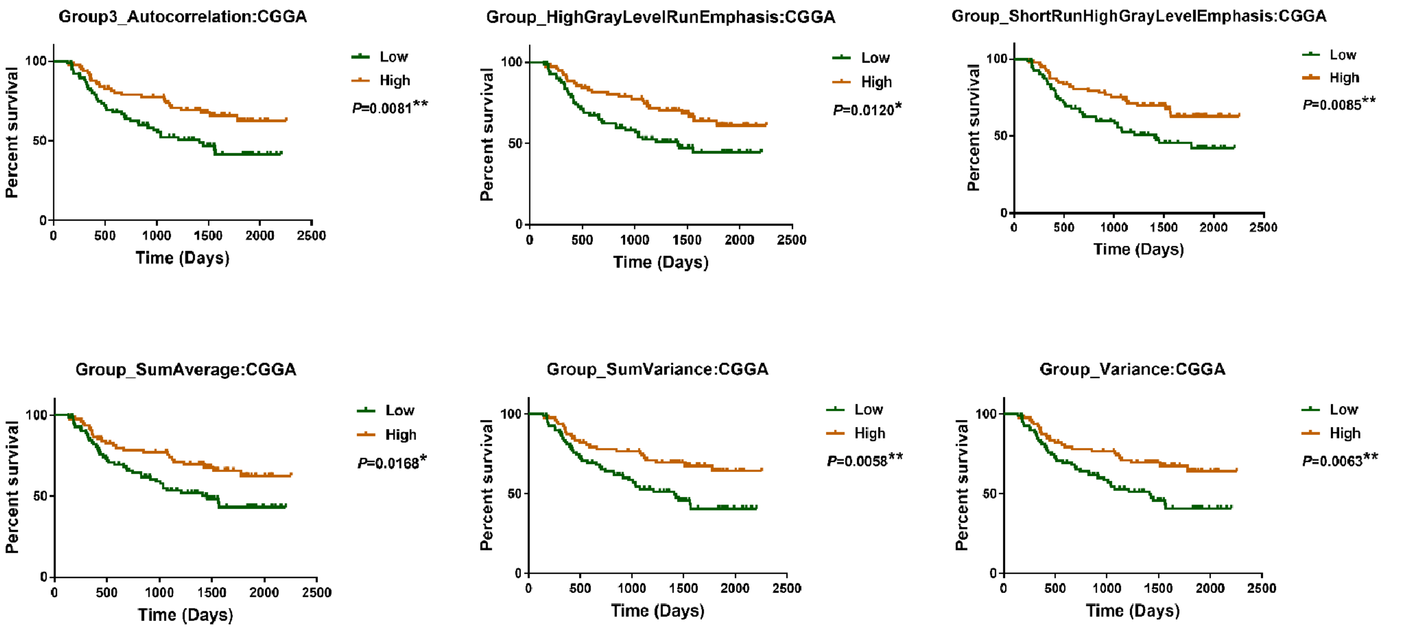

Supplementary Figure 1. Kaplan–Meier plot for overall survival of patients stratified by low- and high-value of each radiomic feature in the validation set.

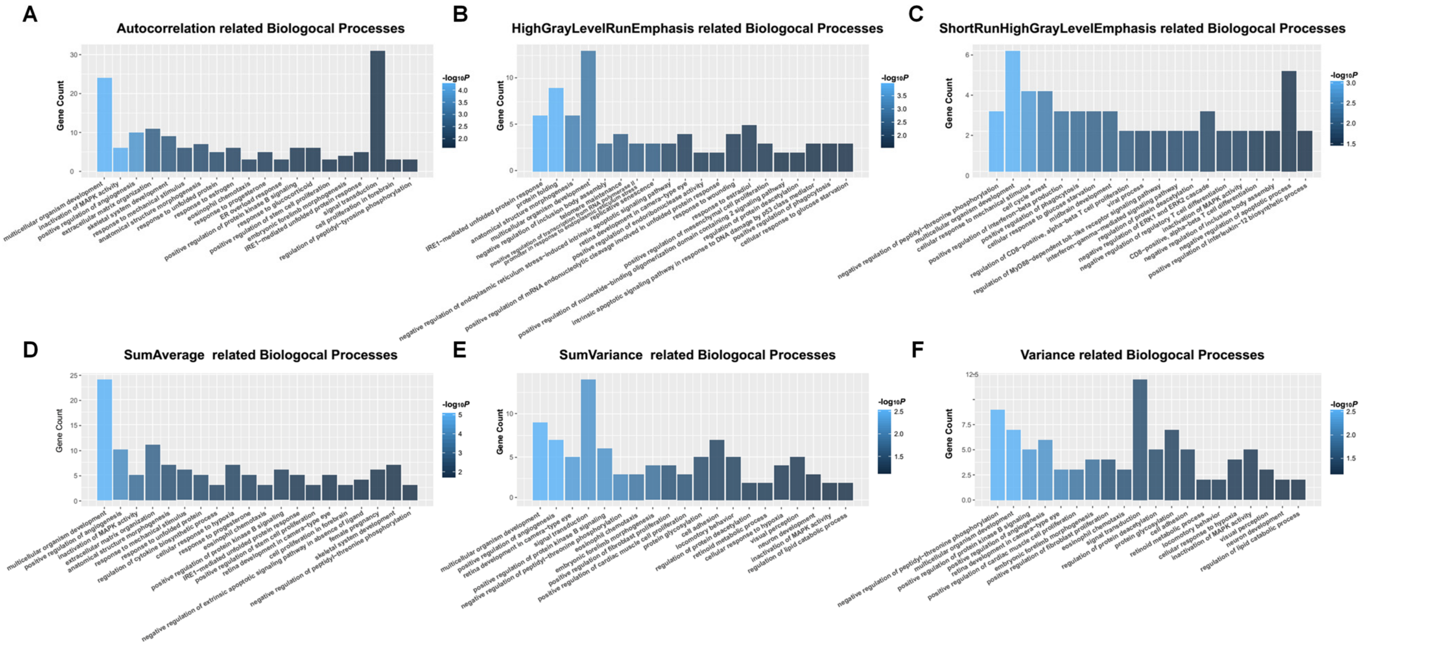

Supplementary Figure 2. Functional annotation of significant radiomic features. Gene ontology analysis revealed a significant association among genes with increased expression in each high-risk radiomic feature and twenty main pathways. Column height: gene counts; point color: enrichment *P* value.

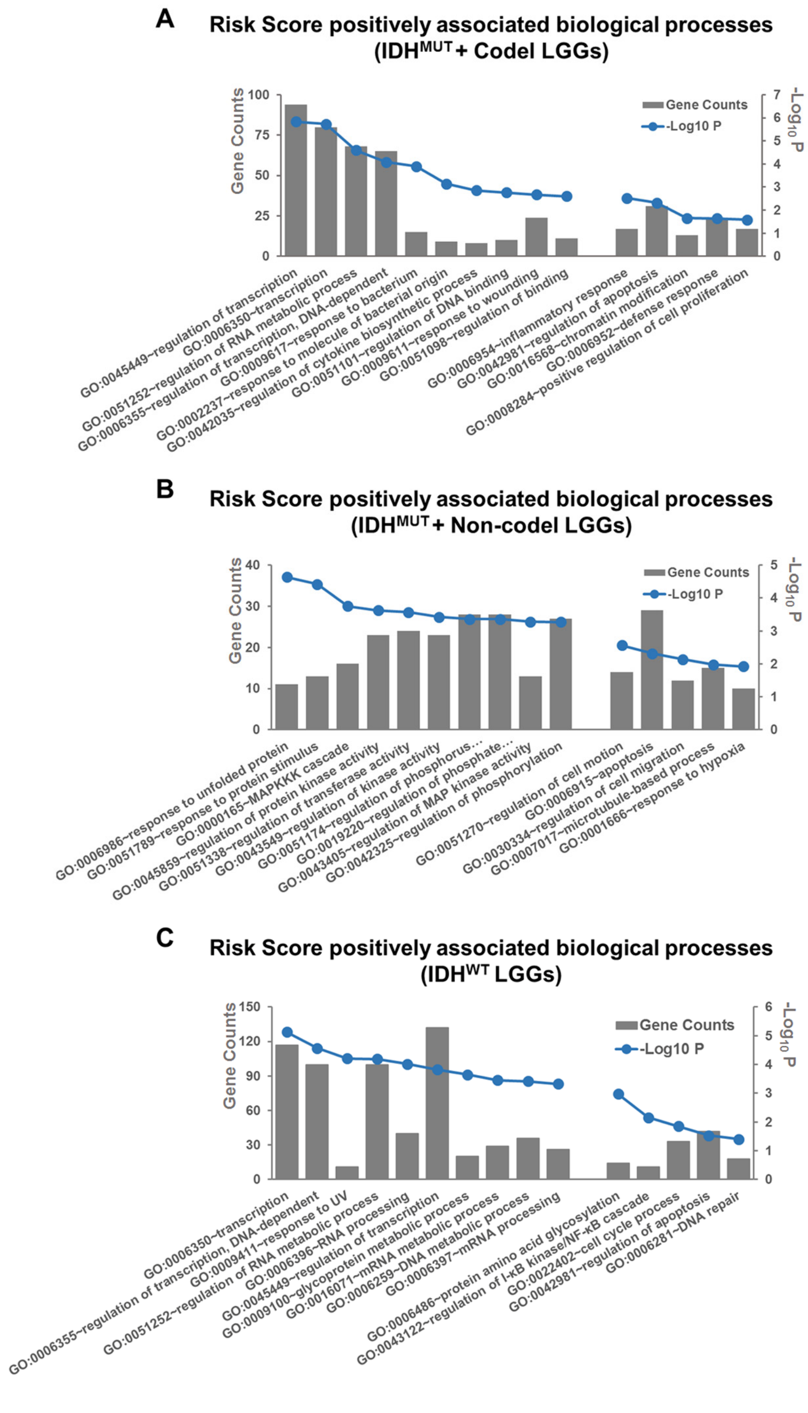

Supplementary Figure 3. The risk score positively associated biological processes among the three molecular classification. IDH mutation and 1p/19q codeletion (**A**), IDH mutation and 1p/19q non-codeletion (**B**), and IDH wild-type (**C**). Column height: gene counts; point color: enrichment *P* value.

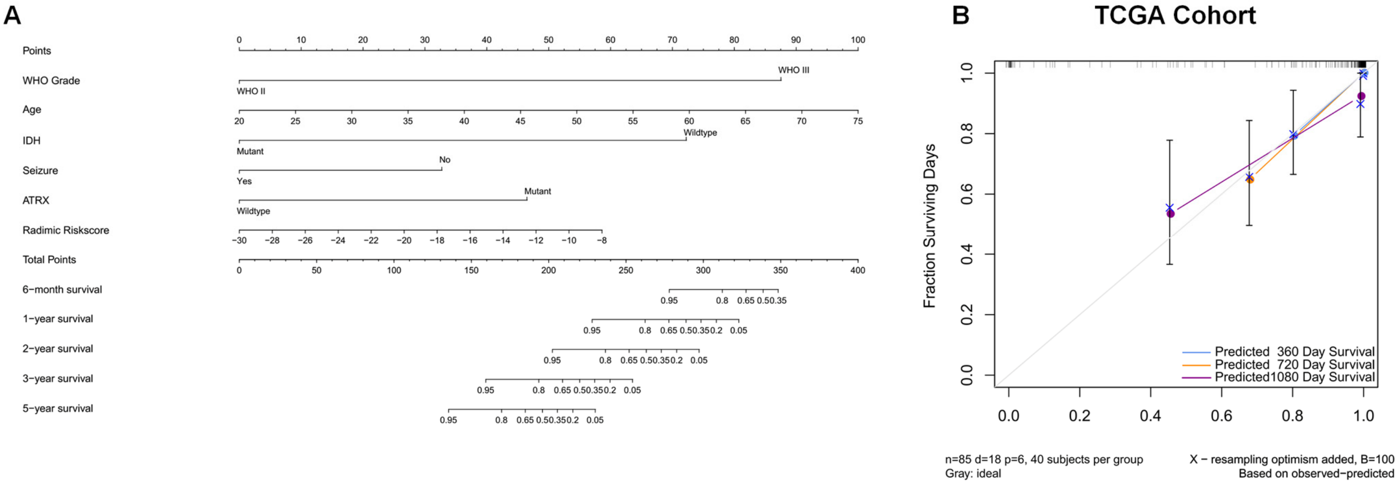

Supplementary Figure 4. A nomogram for predicting overall survival of patients with LGGs (**A**), along with assessment of the model calibration in the training cohort (**B**). After final model selection, radiomic signature, WHO grade, age, IDH status, ATRX status, and seizure were included in the nomogram. The line represents the number of points received for the value of each variable. The sum of these numbers is presented on the total axis, while line drawn down to the survival axis determines the likelihood of a 1-, 2-, 3-, or 5-year survival rate. The calibration curve of the nomogram is also shown. Three colored lines (blue, yellow, and purple) present the performance of the nomogram, with a closer fit to the diagonal line representing a better estimation.
