# Supplementary material for: Radiogenomics of lower-grade gliomas: a radiomic signature as a biological surrogate for survival prediction
Source: Aging (Albany NY). 2018 Oct 22;10(10):2884–99. doi: 10.18632/aging.101594 (PMC6224242; doi:10.18632/aging.101594)
Supplement: Supplementary Tables [file aging-10-101594-s002.pdf]

SUPPLEMENTARY TABLES

Supplementary Table 1. Univariable Cox of radiomic features in TCGA lower grade glioma patients.

Feature name	Interpretation	β	95%CI		HR	*P*
			Lower	Upper		
**Group3_Autocorrelation**	A texture feature derived from Gray-Level Co-Occurrence Matrix based features. Autocorrelation evaluates the linear spatial relationship between texture primitives and measures the coarseness of an image.	-0.007	0.988	0.999	0.993	**0.024**
**Group3_HighGrayLevelRunEmphasis**	One of the texture features derived from Gray-Level Run-Length matrix based features. High Gray-Level Run Emphasis measures the distribution of high gray scale values. This feature is high for the image with high gray scale values.	-0.003	0.996	0.999	0.997	**0.008**
**Group3_ShortRunHighGrayLevelEmphasis**	A texture feature derived from Gray-Level Run-Length matrix based features. Short Run High Gray Level Emphasis describes the complementary metric to the previous one for high gray levels.	-0.005	0.992	0.998	0.995	**0.002**
**Group3_SumAverage**	A texture feature derived from Gray-Level Co-Occurrence Matrix based features. Sum Average measures overall image brightness.	-0.115	0.808	0.983	0.891	**0.022**
**Group3_SumVariance**	One of the texture features derived from Gray-Level Co-Occurrence Matrix based features. It describes the overall voxel intensity value variability of the tumor.	-0.002	0.996	0.999	0.998	**0.024**
**Group3_Variance**	A texture feature derived from Gray-Level Co-Occurrence Matrix based features. Variance is a measure of how much the gray levels differ from the mean.	-0.007	0.987	0.999	0.993	**0.023**

Supplementary Table 2. Genes involved in the "multicellular organism development" group.

Order	GO term	Gene	Pearson correlation coefficient*
1	GO:0007275~multicellular organism development	SPRED1	0.514802938
2	GO:0007275~multicellular organism development	SPRED2	0.479288376
3	GO:0007275~multicellular organism development	SPRY4	0.468034887
4	GO:0007275~multicellular organism development	EDA2R	0.463660169
5	GO:0007275~multicellular organism development	HILS1	0.443597755
6	GO:0007275~multicellular organism development	CREB3L1	0.402457048
7	GO:0007275~multicellular organism development	INVS	0.381701917
8	GO:0007275~multicellular organism development	GCNT2	0.361145274
9	GO:0007275~multicellular organism development	GGN	0.360364448
10	GO:0007275~multicellular organism development	LBH	0.351541368
11	GO:0007275~multicellular organism development	SPRY2	0.349641312
12	GO:0007275~multicellular organism development	EPHA2	0.337424369
13	GO:0007275~multicellular organism development	NFE2	0.336535386
14	GO:0007275~multicellular organism development	HMGA2	0.33571953
15	GO:0007275~multicellular organism development	INSL4	0.333116639
16	GO:0007275~multicellular organism development	OTX1	0.322126755
17	GO:0007275~multicellular organism development	CATSPER3	0.315592995
18	GO:0007275~multicellular organism development	TXNDC2	0.311809689
19	GO:0007275~multicellular organism development	TP53	0.309901514
20	GO:0007275~multicellular organism development	TNFRSF10B	0.309109194
21	GO:0007275~multicellular organism development	EYA4	0.306919931
22	GO:0007275~multicellular organism development	SALL4	0.306853623
23	GO:0007275~multicellular organism development	LIF	0.301635627

*The Pearson correlation coefficient was calculated using the radiomic risk score and the expression of the genes.

Supplementary Table 3. Clinical characteristics of lower grade gliomas in TCGA and CGGA datasets.

	TCGA cohort (n=85)	CGGA cohort (n=148)
**Age (range, median)**	20-74(43)	18-63(38)
**Sex**		
Female	49	54
Male	36	94
**WHO Grade**		
WHO II	45	105
WHO III	40	43
**Seizure**		
Yes	56	89
No	29	59
**IDH status**		
Mutant	65	109
Wildtype	20	39
**ATRX**		
Mutant	34	NA
Wildtype	51	NA
**1p/19q**		
Codeletion	21	22
Non-codeletion	64	47
NA	0	79

NA = Not Available; TCGA = the Cancer Genome Atlas; CGGA = Chinese Glioma Genome Atlas

Supplementary Table 4. Radiomics features extracted from T2-weight magnetic resonance image (n=55).

Groups		
**I. First order statistics (n=14)**		
Energy	Kurtosis	Mean absolute deviation
Entropy	Maximum	Median
Standard deviation	Mean	Minimum
Uniformity	Root mean square (RMS)	Range
Variance	Skewness	
**II. Shape and size based features (n=8)**		
Compactness 1	Spherical disproportion	Surface area
Compactness 2	Sphericity	Surface to volume ratio
Maximum 3D diameter	Volume	
**III. Textural features (n=33)**		
Gray level co-occurrence matrix (GLCM) (n=22)		
Autocorrelation	Energy	Inverse Difference Moment Normalized (IDMN)
Cluster Prominence	Entropy (H)	Inverse Difference Normalized (IDN)
Cluster Shade	Homogeneity 1	Inverse variance
Cluster Tendency	Homogeneity 2	Maximum Probability
Contrast	Informational measure of correlation 1 (IMC1)	Sum average
Correlation	Informational measure of correlation 2 (IMC2)	Sum entropy
Difference entropy	Variance	
Dissimilarity		
Sum variance		
Gray level run-length matrix (GLRLM) (n=11)		
Short Run Emphasis (SRE)	Short Run Low Gray Level Emphasis (SRLGLE)	
Long Run Emphasis (LRE)	Short Run High Gray Level Emphasis (SRHGLE)	
Gray Level Non-Uniformity (GLN)	Long Run Low Gray Level Emphasis (LRLGLE)	
Run Length Non-Uniformity (RLN)	Long Run High Gray Level Emphasis (LRHGLE)	
Run Percentage (RP)	Low Gray Level Run Emphasis (LGLRE)	
	High Gray Level Run Emphasis (HGLRE)	
